# Swimming Training Does Not Affect the Recovery of Femoral Midshaft Structural and Mechanical Properties in Growing Diabetic Rats Treated with Insulin

**DOI:** 10.3390/life11080786

**Published:** 2021-08-03

**Authors:** Gilton de Jesus Gomes, Márcia Ferreira da Silva, Edson da Silva, Ricardo Junqueira Del Carlo, Daise Nunes Queiroz da Cunha, Miguel Araújo Carneiro-Junior, Thales Nicolau Prímola-Gomes, Antônio José Natali

**Affiliations:** 1 Department of Physical Education, Universidade Federal de Viçosa, Viçosa 36570-900, Brazil; gilton.gomes@ufvjm.edu.br (G.d.J.G.); daise.nunes@ufv.br (D.N.Q.d.C.); miguel.junior@ufv.br (M.A.C.-J.); thales.gomes@ufv.br (T.N.P.-G.); 2 Department of Physical Education, Universidade Federal dos Vales do Jequitinhonha e Mucuri, Diamantina 39100-000, Brazil; 3 Sport and Leisure Center, Instituto Federal de Minas Gerais, São João Evangelista 39705-000, Brazil; esportes.sje@ifmg.edu.br; 4 Department of Basic Sciences, Universidade Federal dos Vales do Jequitinhonha e Mucuri, Diamantina 39100-000, Brazil; edson.silva@ufjm.edu.br; 5 Department of Veterinary Medicine, Universidade Federal de Viçosa, Viçosa 36570-900, Brazil; ricarlo@ufv.br

**Keywords:** diabetes, cortical bone, maximum load, tenacity

## Abstract

Background: The effects of swimming training associated with insulin treatment on the cortical bone health in young rats with severe type 1 diabetes remain unclear, although there is evidence of such effects on the cancellous bone. This study examined the effects of swimming training combined with insulin therapy on the femoral midshaft structural and mechanical properties in growing rats with type 1 diabetes. Methods: Male Wistar rats were divided into six groups (*n* = 10): control sedentary, control exercise, diabetic sedentary, diabetic exercise, diabetic sedentary plus insulin and diabetic exercise plus insulin. Diabetic rats received an injection (60 mg/kg body weight) of streptozotocin (STZ). Exercised animals underwent a swimming program for eight weeks. Results: Diabetes induced by STZ decreased the bone mineral content (BMC) and density (BMD), and cortical thickness and maximum load and tenacity in the femoral midshaft. Insulin treatment partially counteracted the damages induced by diabetes on BMC, BMD and cortical thickness and tenacity. Swimming training did not affect the femoral structural and mechanical properties in diabetic rats. The combination of treatments did not potentiate the insulin effects. In conclusion, swimming training does not affect the benefits of insulin treatment on the femoral midshaft structural and mechanical properties in growing rats with severe type 1 diabetes.

## 1. Introduction

Diabetes mellitus is thought to debilitate bone quality, which is associated with damages in the structure and composition of the bone [1,2,3]. The increased bone fragility observed in patients with type 1 diabetes is related to long-term hyperglycemia. This such condition causes nonenzymatic glycation in the bone tissue, and along with augmented cross-links in the collagen matrix it diminishes the formation of new bone [4,5,6]. Impaired bone quality in individuals with type 1 diabetes has been observed at an early age. For instance, young girls with type 1 diabetes [7], and growing female and male rats with streptozotocin (STZ)-induced diabetes exhibit abnormal bone structure (i.e., reduced bone mineral content-BMC and density-BMD) and strength (i.e., reduced resistance to fracture) [8,9,10,11]. It is noteworthy that type 1 diabetes damages bone health even at the early stages of life, and that bone fracture is elevated in diabetic people [3], which negatively affects life quality.

The glycemic level is essentially controlled by insulin, and is known to be associated with regular BMD and reductions in bone resorption in patients with type 1 diabetes [12]. In experimental diabetes, decreases in the BMD in young rats caused by STZ may be avoided by insulin treatment [13]. Moreover, the damages caused by STZ in the cancellous bone structure and biomechanical function is ameliorated by insulin in young rats [8,11].

Physical exercise promotes benefits to bone health [14], as it counterbalances bone fragility because of its inherent osteogenic properties. Swimming training has been shown, by our group [10] and elsewhere [15,16], to enhance cancellous bone (i.e., femoral neck) structure and strength in young rats with type 1 diabetes; however, the low impact of swimming training, and, hence, its osteogenic properties are controversial [17,18]. Considering that cortical bone (i.e., femoral shaft) is less responsive than cancellous bone to the mechanical load imposed by exercise [19], we hypothesized that swimming training does not affect the cortical bone structural and mechanical properties in young rats with STZ-induced severe type 1 diabetes, even when associated with insulin treatment. Thus, the aim of this study was to examine the effects of swimming training combined with insulin therapy on the femoral cortical bone structural and mechanical properties in growing rats with type 1 diabetes.

## 2. Materials and Methods

Male Wistar rats (age: 40 days; body weight: 89.71 ± 2.34 g) were allocated into one of the following experimental groups (*n* = 10 animals per group): control sedentary (CS), control exercise (CE), diabetic sedentary (DS), diabetic exercise (DE), diabetic sedentary plus insulin (DSI) and exercise plus insulin (DEI). All animals were kept in a room (humidity, ~65%), where the light-dark cycle (12/12 h) and temperature (~22 °C) were controlled. The rats had water and commercial rodent chow ad libitum. The experiments were carried out in accordance with the international ethical standards, and had the approval of the ethics committee on animal use of the Universidade Federal de Viçosa, Brazil (protocol n. 51/2011).

The rats in the diabetic groups received one injection intraperitonially (60 mg/kg of body weight (BW)) of STZ (Sigma-Aldrich, St. Louis, MO, USA), diluted in 1.0 mL of a buffer solution (sodium citrate 0.1 M, pH 4.5). The rats in control groups received the same dosage sodium citrate. Type 1 diabetes was confirmed one week after STZ injection by measuring the blood glucose (BG) levels in the animals (One Touch Ultra, Johnson & Johnson, Chihuahua, Mexico) at rest. The animals exhibiting fasting hyperglycemia (i.e., BG levels ≥ 300 mg/dL) were considered diabetic [13]. Both STZ injection and BG measurement were performed after 12 h of fasting. Blood glucose was monitored weekly.

One week after hyperglycemia, animals from DE, DEI and CE groups underwent the swimming training, as previously described [10]. In summary, the animals exercised 90 min/day, 5 days/week for 8 weeks. During the first week, the animals started swimming for 10 min/day, then this duration was augmented by 10 min/day without load. During the second week, the duration of the exercise was increased by 10 min/day until it reached 90 min without load. This protocol was repeated in the third week. Then, during the fourth, fifth, sixth, seventh and eighth weeks, the rats swam 90 min/day, and an additional load of 1% of BW per week was attached to the animal.

The insulin treatment in the DSI and DEI groups consisted of 2–4 U/day/rat (i.e., 1U per 60 g of BW) during the experimental period of 8 weeks. During the first week, a subcutaneous injection of 2 units was given 6 h after the swimming session. The insulin dose was altered to keep the BG at ~300 mg/dL (i.e., severe type 1 diabetes).

Forty-eight hours after the last swimming session, at euthanasia, the right and left femurs were dissected. For the histomorphometry, the left femur was immersed in neutral buffered formalin (10%). The right femur was used for the femoral midshaft mechanical properties measurement. For this, it was removed and stored in saline solution in a freezer (−20 °C).

After decalcification and dehydration, transversal sections (5 µm thick) were cut from the midshaft region of the left femur by using a microtome (Leica 2065, Leica Biosystems, Wetzlar, Germany). The sections were then prepared for histology slides as described in a previous study [11]. To measure the cortical bone thickness, the slides were tinged with hematoxylin and eosin.

To measure the cortical bone thickness, two images (100×) in different fields from each rat were obtained by using a photomicroscope (Olympus Biological CX31, Olympus, Tokyo, Japan), supplied with the software analySIS^®^ getIT (Olympus Soft Imaging Solutions GmbH, Olympus, Tokyo, Japan). The Image-Pro Plus software, version 4.5.0.29 (Media Cybernetics, Rockville, MD, USA) was used to analyze the images. We used the mean of 20 measures per animal to find the cortical bone thickness.

The femoral BMC and BMD, and midshaft mechanical properties were measured in the right femur. The femur was thawed until reaching room temperature (23 °C) in a saline solution (~2 h). The femur was then scanned using dual energy X-ray absorptiometry, to determine BMC and BMD. In brief, the femur was placed in a Plexiglas container filled with deionized water and scanned using Lunar DPX Alpha (GE Healthcare, Chicago, IL, USA), equipped with small-animal software. The femoral area was measured after selecting the specific area of interest (whole femur) and drawing a box around it. After that, the femoral midshaft mechanical properties were measured in the right femurs, by using a three-point test as described elsewhere [20]. Briefly, we settled the femur transversally on two supports on a universal testing machine (EMIC, DL 3000, São José dos Pinhais, Brazil), containing a load cell (2000 N) regulated by a computer. The cell force was imposed downward on the femoral midshaft at a steady velocity of 0.5 mm/min until the femur fractured. A frequency of 60 Hz was used to record data, that were transformed to obtain a curve of load displacement. From this curve, the maximum load, stiffness and tenacity were calculated. The femoral midshaft mechanical properties were normalized by the BW, as diabetic rats exhibited lower BW than control rats.

The normality of observations, and homogeneity of variance between groups were checked. To compare data for initial and final BW, we employed the paired Student *t* test. To compare data for BG at different times we used a two-way repeated-measures analysis of variance (ANOVA), followed by the Tukey’s post hoc test. Data for structural and mechanical properties were compared using the two-way ANOVA, followed by the Tukey’s post hoc test.

## 3. Results

### 3.1. Body Weight and Blood Glucose

The control rats presented a higher final BW than the diabetic rats with no insulin treatment (Table 1). The insulin treatment partially restored the final BW of diabetic rats. Diabetic rats treated with insulin and nontreated rats had significantly different BW. Swimming significantly reduced the final BW in control rats, though it did not in diabetic rats. Combining these treatments did not affect the final BW.

The STZ injection augmented BG levels within 7 days (~365 mg/dL), and the final BG reached ~500 mg/dL in rats with no insulin treatment. The insulin treatment diminished the final BG to ~320 mg/dL in both the sedentary and trained diabetic rats. However, neither swimming training nor the combination of treatments changed BG levels. 

### 3.2. Femoral Midshaft Structural Properties

**Figure 1:**
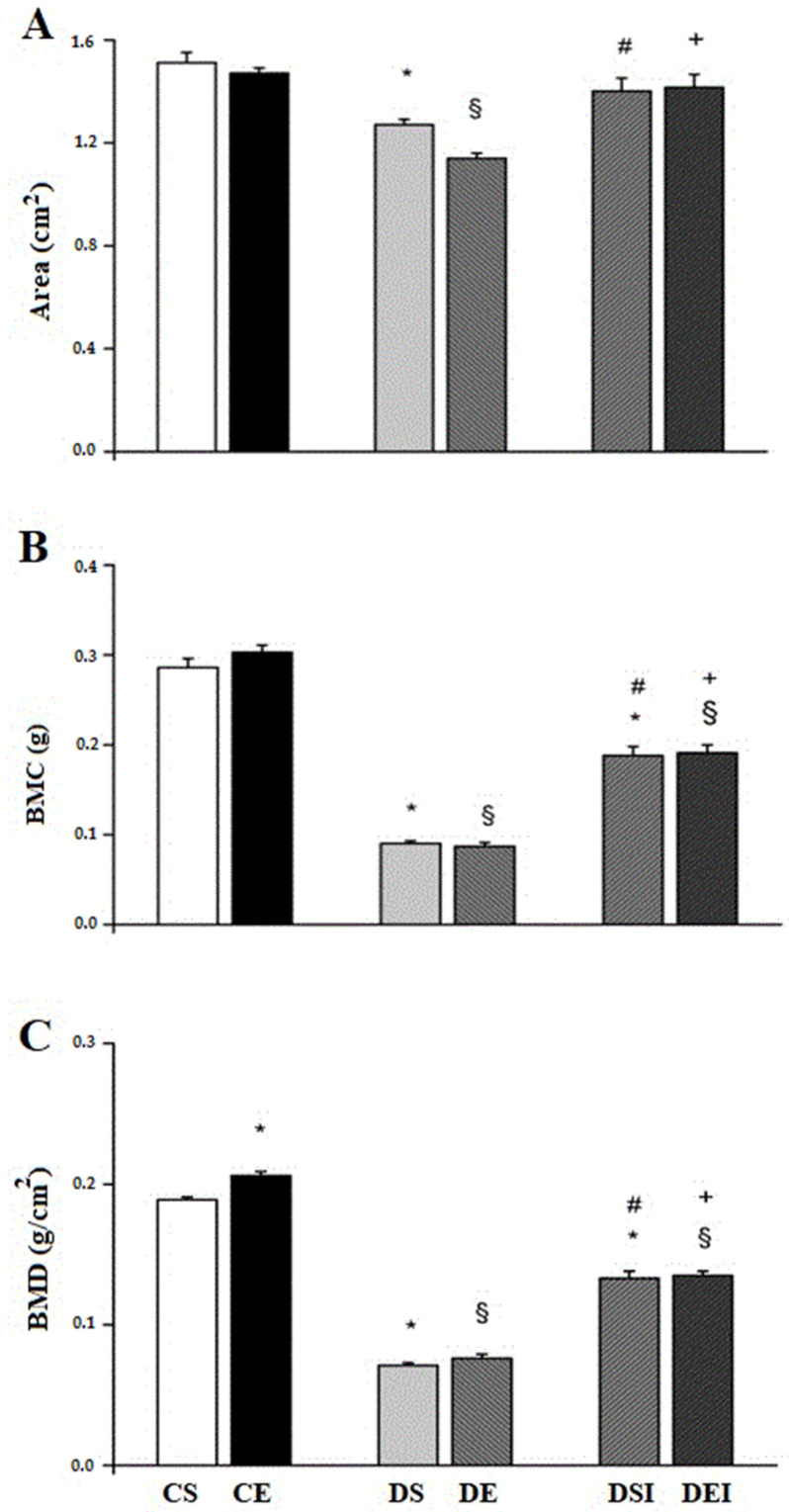
Bone mineral content and density. (**A**) Femoral area. (**B**) Bone mineral content. (**C**) Bone mineral density. Data are means ± SEM of 10 animals in each group. CS, control sedentary. CE, control exercise. DS, diabetic sedentary. DE, diabetic exercise. DSI, diabetic sedentary insulin. DEI, diabetic exercise insulin. * different from de CS. **^§^** different from CE. ^#^ different from DS. ^+^ different from DE (*p* ≤ 0.05).

The rats from the CS group exhibited higher (16%) femoral area than those in the DS group (Figure 1A). In addition, the femoral area was higher (23%) in the CE group, compared with the DE group. The femoral area in diabetic rats treated with insulin was not significantly different from that in the control groups (CE = DEI and CS = DSI). Animals under insulin treatment had a femoral area higher than those not treated with insulin (DEI > DE (20%) and DSI > DS (10%)). However, insulin treatment did not affect the femoral area either in diabetic or control rats.

The BMC was lower (67%) in the DS group than in the CS group (Figure 1B). Similarly, the BMC was lower (72%) in the DE group, compared with the CE group. The animals treated with insulin presented lower BMC than control animals (DEI < CE (37%) and DSI < CS (34%)). Nonetheless, rats treated with insulin showed higher BMC, compared with animals not treated with insulin (DEI > DE (58%) and DSI > DS (52%)). The applied swimming training did not change the BMC in either diabetic or control animals.

Regarding BMD, rats in the CE group presented a higher BMD (9%), compared with those in the CS group (Figure 1C). Nevertheless, animals in the DE and DEI groups showed BMD values similar to those in their respective controls, DS and DSI. Swimming training did not affect the BMD in diabetic animals. Rats in control groups had higher values for BMD than those in diabetic groups without insulin treatment (CE > DE (64%) and CS > DS (3%)). The DEI group exhibited higher (49%) BMD values than the DE group, and the DSI group had higher (47%) BMD values compared to the DS group.

**Figure 2:**
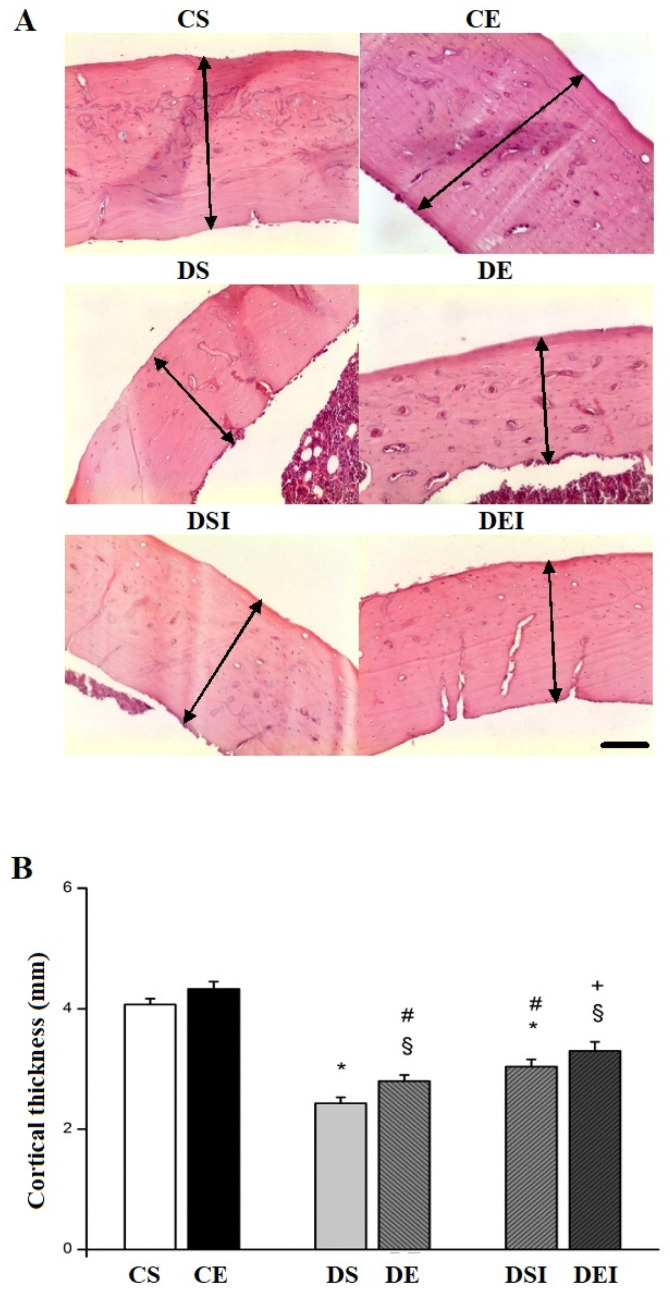
Femoral cortical bone thickness. (**A**) Representative photomicrographs of the femoral midshaft of the animals in the experimental groups. Black arrows: cortical thickness. Hematoxylin and eosin staining. 10×. Bar: 100 μm. (**B**) Mean data for cortical thickness. Data in panel B are means ± SEM. CS, control sedentary. CE, control exercise. DS, diabetic sedentary. DE, diabetic exercise. DSI, diabetic sedentary insulin. DEI, diabetic exercise insulin. * different from CS. **^§^** different from CE. ^#^ different from DS. ^+^ different from DE (*p* ≤ 0.05).

Concerning the femoral midshaft morphology, Figure 2A illustrates the differences between groups for midshaft thickness. The CE group showed higher midshaft thickness than the DE (34%) and DEI (23%) groups (Figure 2B). Similarly, the CS group had a higher thickness compared with the DS (40%) and DSI (25%) groups. Insulin treatment mitigated the midshaft thickness reduction, as DEI (15%) and DSI (20%) groups presented higher cortical thickness than the DE and DS groups, respectively. The swimming training regime employed did not affect the femoral midshaft thickness.

### 3.3. Femoral Midshaft Mechanical Properties

**Figure 3:**
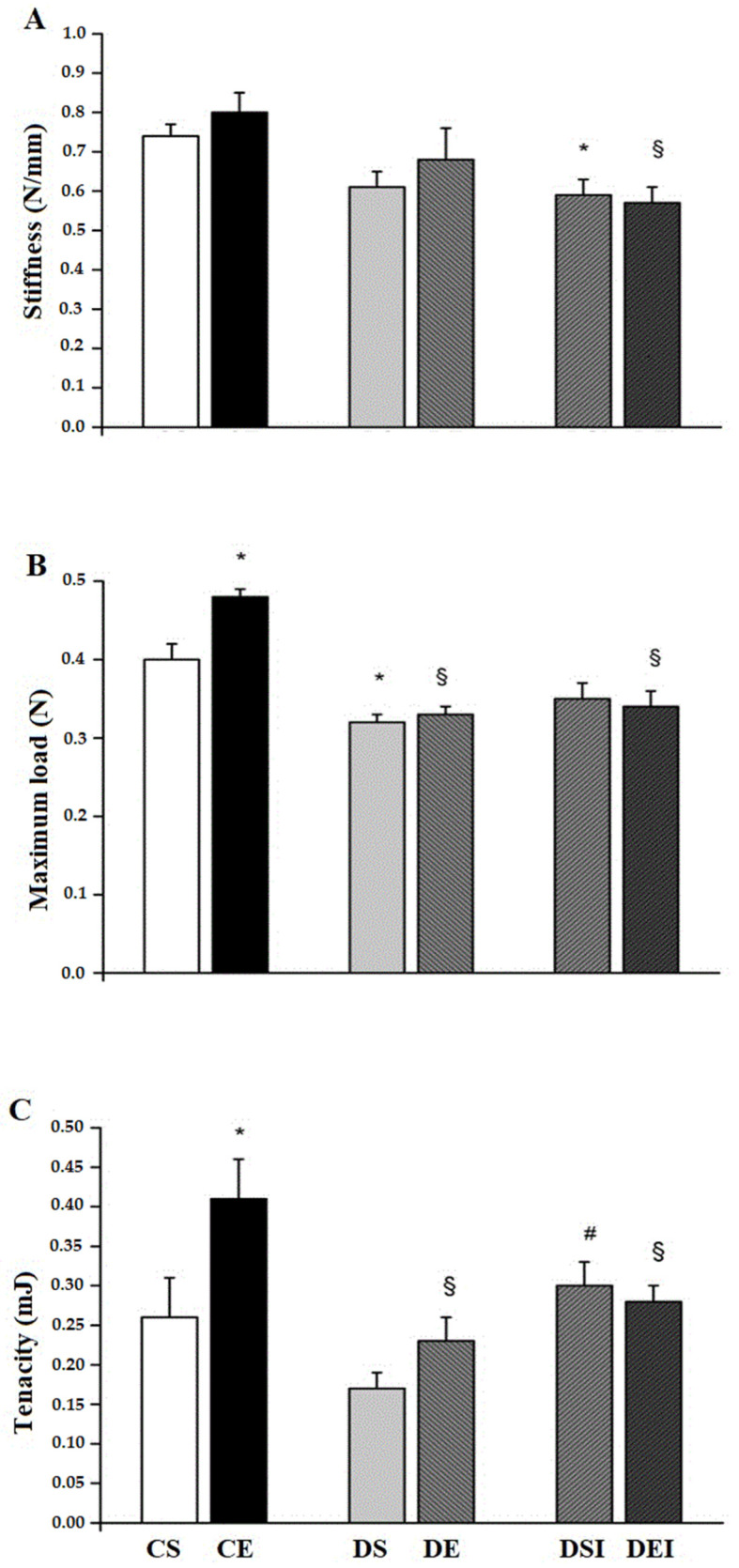
Femoral midshaft mechanical properties. (**A**) Stiffness. (**B**) Maximum load. (**C**) Tenacity. Data are means ± SEM. CS, control sedentary. CE, control exercise. DS. diabetic sedentary. DE, diabetic exercise. DSI, diabetic sedentary insulin. DEI, diabetic exercise insulin. * different from CS. **^§^** different from CE. ^#^ different from DS. (*p* ≤ 0.05).

Streptozotocin-induced diabetes reduced the femoral midshaft stiffness. This such effect reached statistical significance in rats under insulin treatment, as they exhibited lower values (~32%) than control animals (Figure 3A). 

The femoral midshaft maximum load was lower (~20%) in rats in the DS group, compared with those from the CS group (Figure 3B). Animals in the CE group had a higher maximum load (~32%) than those in the DE and DEI groups. The swimming training increased the maximum load in control rats (CE > CS; ~18%) and had no effect on diabetic rats. However, the combination of treatments did not affect the femoral midshaft maximum load.

The femoral midshaft tenacity was significantly lower in rats from DE (~46%) and DEI (~32%) groups, than in those from the CE group. Animals in the DSI group showed higher (~53%) tenacity compared with rats from the DS group. Swimming training alone increased the femoral midshaft tenacity in the control rats only (CE > CS; ~36%). However, the combination of treatments did not affect the femoral midshaft tenacity.

## 4. Discussion

This study examined the effects of swimming training combined with insulin therapy on the femoral midshaft structural and mechanical properties in growing diabetic rats. We demonstrate that severe type 1 diabetes induced by STZ reduced the femoral BMC, BMD and the area and midshaft thickness. Reductions in bone mass in individuals with type 1 diabetes has been shown to result from the diminished secretion and/or action of insulin, which is associated with the formation of advanced glycation end-products (AGEs), induced by oxidative stress and hyperglycemia. Therefore, the formation of new bone is impaired by the unbalanced osteoblast and osteoclast activities, favoring the osteoclast’s activity [21,22]. Moreover, it has been suggested in experimental type 1 diabetes that active osteoblasts may return to inactive bone-lining cells, leading to an impaired proliferation of preosteoblast cells [23].

The insulin treatment employed here partially recovered the femoral BMC, BMD and midshaft thickness in diabetic rats, which was also reflected in the recovery of the femoral area. Insulin is reported to exert an anabolic effect on the bone and regulate bone turnover [24]. As insulin reduces the BG levels, nonenzymatic glycation is diminished, and as a consequence there is less AGEs formation. Moreover, the formation and mineralization of cross-links in the bone collagen matrix is shortened by insulin [4].

Although swimming training alone increased the BMD in control animals, it did not counterbalance the damages associated with STZ-induced diabetes (i.e., reduced femoral BMC, cortical thickness and area). These results suggest that the applied swimming regime did not provide sufficient osteogenic stimuli. Despite this, nonweight-bearing exercise is reported to positively influence the skeleton by promoting increases in muscle mass and strength, which by means of mechanoreceptors triggers osteogenic signals in the bone [12]. The bone deposition is also observed in response to this type of exercise, since it changes the hormonal levels [25]. Nevertheless, the combination of treatments did not potentiate the effects of insulin on the femoral structural properties (i.e., BMC, BMD, cortical thickness and area) in diabetic rats.

The severe diabetes induced by STZ worsened the femoral midshaft mechanical properties (i.e., reduced maximum load and tenacity). The maximum load reflects bone strength, and bone mass and collagen are known to underlie such a property. Thus, it is conceivable that the diminished femoral area, BMC, BMD and cortical thickness resulted in reduced bone resistance to fracture in the diabetic rats. Although it was not measured in the present study, reduced collagen content has been reported in this model of severe diabetes [10]. Concerning the low tenacity observed in diabetic rats, it is possible that a reduction in the femoral collagen in these rats has contributed to such damage, since this measure relies on the unmineralized matrix [26]. Reduced bone strength in type 1 diabetes may result from decreased bone collagen, and, hence, decreased bone mass [4]. It is important to mention that bone microarchitecture and microdamage also debilitates bone integrity in experimental diabetes [27].

The applied treatment with insulin enhanced the femoral midshaft tenacity in diabetic rats. As bone tenacity is determined primarily by the unmineralized matrix [26], it is conceivable that the observed insulin benefit to femoral tenacity is due to an increase in the femoral collagen content promoted by insulin in this model of experimental diabetes, as reported previously by our group [10].

Although swimming training increased the femoral maximum load and tenacity in control non-diabetic rats, no effect of exercise on femoral mechanical properties was found in diabetic rats, either with or without insulin treatment. Thus, swimming training did not potentiate the isolated effect of insulin treatment (i.e., combined treatment) on the mechanical properties. We have demonstrated in a previous study [10] that swimming training did not affect the femoral neck mechanical properties in diabetic rats without insulin treatment; nonetheless, it potentiated the effects of insulin therapy. In this sense, the findings of the present study reinforce the differences between femoral cancellous and cortical bone, either with or without insulin therapy, in responding to exercise. When comparing the bone types, our results suggest that the swimming training load, via muscle contraction, is higher on the neck than on the midshaft region of the femur, which reflects more osteogenic exercise effects on the cancellous bone. Thus, exercise leads to greater changes in the bone mass [10,28], content [10,29] and orientation of collagen fibers [30] in the neck than in the shaft region. Our results also strengthen the concept that insulin is crucial for the bone metabolism regulation [4,21,22,23,24], independent of the bone type, as the absence of insulin damages the structural and mechanical properties of the femoral neck and shaft, both in exercised and non-exercised diabetic rats, as demonstrated here and in the previous studies of our group [10,11].

We did not measure serum and urinary bone markers and intestinal calcium absorption, which may affect the bone mass in diabetic rats [31]. Moreover, insulin concentrations were not assessed, although the measurement of BG levels indicates the insulin deficiency. Thus, we consider the absence of such measures as study limitations.

It is noteworthy that bone loading patterns are different between humans and rats. Despite this, the effects of exercise and insulin therapy on diabetic rat cortical bone may be of clinical relevance because of the evident osteopenia and risk for fracture in patients with type 1 diabetes. Furthermore, even though reduced bone mass was found in young subjects with moderate type 1 diabetes with metabolic control [32], the findings in the present study are related to severe diabetes (i.e., BG ≥ 300 mg/dL), thus, it is difficult to generalize to patients treated with insulin.

## 5. Conclusions

In contrast to the effects of swimming training on the femoral neck of growing rats with severe STZ-induced type 1 diabetes under insulin therapy, the benefits of insulin treatment on the femoral midshaft structural and mechanical properties were not affected by swimming training in this model.

## Figures and Tables

**Table 1:** Body weight (BW) and blood glucose (BG) over the experimental period.

Groups	N	Initial BW (g)	Final BW (g)	Initial BG (mg/dL)	BG 7 Days Post-STZ Injection (mg/dL)	Final BG (mg/dL)
CS	10	88.61 ± 1.12	347.11 ± 6.74 ^§a^	71.52 ± 1.89	-	94.3 ± 6.41 ^b^
CE	10	90.80 ± 2.54	281.32 ± 11.14 *^a^	72.20 ± 5.66	-	80.0 ± 3.17
DS	10	89.52 ± 1.82	160.40 ± 3.74 *^a^	72.10 ± 5.78	409.00 ± 28.72 ^b^	573.3 ± 12.13 *^bc^
DE	10	88.91 ± 3.81	164.11± 12.56 ^§a^	73.41 ± 4.26	367.61 ± 19.46 ^b^	504.4 ± 30.43 ^§bc^
DSI	10	91.80 ± 3.09	240.31 ± 6.17 *^#a^	62.12 ± 1.08	392.12 ± 16.85 ^b^	312.1 ± 44.20 *^#b^
DEI	10	88.60 ± 1.69	254.72 ± 6.27 ^§+a^	69.13 ± 3.11	365.02 ± 16.18 ^b^	339.6 ± 50.22 ^§+b^

Data are expressed as the means ± SEM of 10 rats in each group. N: number of animals; CS: control sedentary; CE: control exercise; DS: diabetic sedentary; DE: diabetic exercise; DSI: diabetic sedentary plus insulin; DEI: diabetic exercise plus insulin. * different from CS, **^§^** different from CE, ^#^ different from DS and ^+^ different from DE (in the same column; *p* ≤ 0.05). ^a^ different from initial BW, ^b^ different from initial BG and ^c^ different from BG 7 days post STZ (*p* ≤ 0.05).

## Data Availability

Not applicable.

## References

[1] Napoli N., Chandran M., Pierroz D.D., Abrahamsen B., Schwartz A.V., Ferrari S.L. (2017). Mechanisms of diabetes mellitus-induced bone fragility. Nat. Rev. Endocrinol..

[2] Sellmeyer D.E., Civitelli R., Hofbauer L.C., Khosla S., Lecka-Czernik B., Schwartz A.V. (2016). Skeletal metabolism, fracture risk, and fracture outcomes in type 1 and type 2 diabetes. Diabetes.

[3] Vestergaard P., Rejnmark L., Mosekilde L. (2009). Diabetes and its complications and their relationship with risk of fractures in type 1 and 2 diabetes. Calcif. Tissue Int..

[4] Saito M., Fujii K., Mori Y., Marumo K. (2006). Role of collagen enzymatic and glycation induced cross-links as a determinant of bone quality in spontaneously diabetic BN/Kob rats. Osteoporos Int..

[5] Saito M., Marumo K. (2010). Collagen cross-links as a determinant of bone quality: A possible explanation for bone fragility in aging, osteoporosis, and diabetes mellitus. Osteoporos Int..

[6] Nyman J.S., Even J.L., Jo C., Herbert E.G., Murry M.R., Cockrell G.E., Wahl E.C., Bunn R.C., Lupkin C.K., Fowlkes J.L. (2011). Increasing duration of type 1 diabetes perturbs the strength-structure relationship and increases brittleness of bone. Bone.

[7] Soto N., Pruzzob R., Eyzaguirrec F., Iñiguez G., López P., Mohr J., Perez-Bravo F., Cassorla F., Codner E. (2011). Bone mass and sex steroids in postmenarcheal adolescents and adult women with type 1 diabetes mellitus. J. Diabetes Complicat..

[8] Hou J.C.H., Zernicke R.F., Barnard R.J. (1993). Effects of severe diabetes and insulin on the femoral neck of the immature rat. J. Orthop. Res..

[9] Hofbauer L.C., Brueck C.C., Singh S.K., Dobnig H. (2007). Osteoporosis in patients with diabetes mellitus. J. Bone Min. Res..

[10] Gomes G.J., Del Carlo R.J., da Silva M.F., Cunha D.N.Q., da Silva E., Silva K.A., Carneiro-Junior M.A., Prímola-Gomes T.N., Natali A.J. (2019). Swimming training potentiates the recovery of femoral neck strength in young diabetic rats under insulin therapy. Clinics.

[11] Silva K.A., Del Carlo R.J., Matta S.L.P., Louzada M.J.Q., Rodrigues A.C., Silva M.F., Drummond L.R., Castro C.A., Silva C.H.O., Natali A.J. (2014). Effects of swimming training on the femoral neck strength in growing rats with untreated streptozotocin-induced diabetes. Int. Sport Med J..

[12] Hamrick M.W., Samaddar T., Pennington C., McCormick J. (2006). Increased muscle mass with myostatin deficiency improves gains in bone strength with exercise. J. Bone Miner. Res..

[13] Erdal N., Gurgul S., Demirel C., Yildiz A. (2012). The effect of insulin therapy on biomechanical deterioration of bone in streptozotocin (STZ)-induced type 1 diabetes mellitus in rats. Diabetes Res. Clin. Pract..

[14] Beck B.R., Dalyb R.M., Singhc M.A.F., Taaffed D.R. (2017). Exercise and Sports Science Australia (ESSA) position statement on exercise prescription for the prevention and management of osteoporosis. J. Sci. Med. Sport.

[15] Ju Y.I., Sone T., Ohnaru K., Tanaka K., Fukunaga M. (2015). Effect of swimming exercise on three-dimensional trabecular bone microarchitecture in ovariectomized rats. J. Appl. Physiol..

[16] Kang Y.-S., Kim S.-H., Kim J.-C. (2017). Effects of swimming exercise on high-fat diet-induced low bone mineral density and trabecular bone microstructure in rats. J. Exerc. Nutr. Biochem..

[17] Gomez-Bruton A., Gonzalez-Aguero A., Gomez-Cabello A., Casajus J.A., Vicente-Rodriguez G. (2013). Is bone tissue really affected by swimming? A systematic review. PLoS ONE.

[18] Portier H., Benaitreau D., Pallu S. (2020). Does physical exercise always improve bone quality in rats?. Life.

[19] Borer K.T. (2005). Physical Activity in the prevention and amelioration of osteoporosis. Sports Med..

[20] Mosekilde L., Thomsen J.S., Orhii P.B., McCarter R.J., Mejia W., Kalu D.N. (1999). Additive effect of voluntary exercise and growth hormone treatment on bone strength assessed at four different skeletal sites in an aged rat model. Bone.

[21] Duarte V.M.G., Ramos A.M.O., Rezende L.A., Macedo U.B.O., Brandão-Neto J., Almeida M.G., Rezende A.A. (2005). Osteopenia: A bone disorder associated with diabetes mellitus. J. Bone Miner. Metab..

[22] McCarthy A.D., Uemura T., Etcheverry S.B., Cortizo A.M. (2004). Advanced glycation endproducts interefere with integrin-mediated osteoblastic attachment to a type-I collagen matrix. Int. J. Biochem. Cell Biol..

[23] Weiss R.E., Reddy A.H. (1980). Influence of experimental diabetes and insulin on matrix-induced cartilage and bone differentiation. Am. J. Physiol. Endocrinol. Metab..

[24] Yang J., Zhang X., Wang W., Liu J. (2010). Insulin stimulates osteoblast proliferation and differentiation through ERK and PI3K in MG-63 cells. Cell Biochem. Funct..

[25] Maïmoun L., Sultan C. (2009). Effect of physical activity on calcium homeostasis and calciotropic hormones: A review. Calcif. Tissue Int..

[26] Boivin G., Bala Y., Doublier A., Farlay D., Ste-Marie L.G., Meunier P.J., Delmas P.D. (2008). The role of mineralization and organic matrix in the microhardness of bone tissue from controls and osteoporotic patients. Bone.

[27] Kohn D.H., Sahar N.D., Wallace J.M., Golcuk K., Morris M.D. (2009). Exercise alters mineral and matrix composition in the absence of adding new bone. Cells Tissues Organs..

[28] Robling A.G., Castillo A.B., Turner C.H. (2006). Biomechanical and molecular regulation of bone remodeling. Annu. Rev. Biomed. Eng..

[29] Saino H., Luther F., Carter D.H., Natali A.J., Turner D.L., Shahtaheri S.M., Aaron J.E. (2003). Evidence for an extensive collagen Type III proximal domain in the rat fe.mur II. Expansion with exercise. Bone.

[30] Guadalupe-Grau A., Fuentes T., Guerra B., Calbet A.L. (2009). Exercise and Bone Mass in Adults. Sports Med..

[31] Blakytny R., Spraul M., Jude E.B. (2001). The Diabetic Bone: A Cellular and Molecular Perspective. Int. J. Lower Ext. Wounds.

[32] Moyer-Mileur L.J., Slater H., Jordan K.C., Murray M.A. (2008). IGF-1 and IGF-binding proteins and bone mass, geometry, and strength: Relation to metabolic control in adolescent girls with type 1 diabetes. J. Bone Miner. Res..

